# Vasoaktive Substanzen im septischen Schock – individualisierte Strategien

**DOI:** 10.1007/s00063-025-01272-x

**Published:** 2025-04-24

**Authors:** Georg Franz Lehner, Timo Mayerhöfer, Fabian Perschinka, Bernhard Benda, Michael Joannidis

**Affiliations:** Gemeinsame Einrichtung für Internistische Notfall- und Intensivmedizin, Innere Medizin 1, Department für Innere Medizin, Medizinische Universität Innsbruck, Anichstraße 35, 6020 Innsbruck, Österreich

**Keywords:** Vasopressoren, Vasopressin, β‑Blocker, Sepsis, Noradrenalin, Angiotensin II, Vasopressor agents, Vasopressin, Beta-blockers, Sepsis, Noradrenaline, Angiotensin II

## Abstract

Die Stabilisierung des Kreislaufs und der Erhalt der Organperfusion sind zentrale Elemente in der Therapie des septischen Schocks. Hierfür werden Flüssigkeit und vasoaktive Substanzen verabreicht. Aktuelle Leitlinien empfehlen Noradrenalin als primäre vasoaktive Substanz. Bei hohem Noradrenalinbedarf wird die zusätzliche Verwendung nichtadrenerger Vasopressoren empfohlen. Zudem gibt es Evidenz, dass ein frühzeitiger Einsatz komplementärer vasoaktiver Substanzen von Vorteil sein kann. Ein solches Regime, im Sinne eines Breitspektrumvasopressorkonzepts, erscheint auch aus pathophysiologischer Perspektive schlüssig. Darüber hinaus legen Post-hoc-Analysen von Studien mit Vasopressin oder Angiotensin II nahe, dass spezifische Subphänotypen besonders von einzelnen vasoaktiven Substanzen profitieren könnten. Die adjunktive Therapie mit Hydrokortison und Fludrokortison kann das Ansprechen auf Vasopressoren verbessern und die Mortalität senken. Bei kardialer Dysfunktion wird primär ein Therapieversuch mit Dobutamin oder eine Umstellung von Noradrenalin auf Adrenalin empfohlen. Zur Verstärkung der inodilatatorischen Effekte können Milrinon oder Levosimendan für manche Patient:innen eine zusätzliche Therapieoptionen darstellen. Kurzwirksame β‑Blocker sind zwar nicht Teil der Standardtherapie im septischen Schock, können aber in Einzelfällen bei inadäquat hoher Sinustachykardie oder atrialen Tachyarrhythmien zur hämodynamischen Verbesserungen beitragen. Basierend auf pathophysiologischen Überlegungen und der derzeit verfügbaren Evidenz kann der gezielte Einsatz spezifischer vasoaktiver Substanzen bei bestimmten Subphänotypen gerechtfertigt sein. So könnte auch eine initiale Breitspektrumvasopressortherapie unter Berücksichtigung von Biomarkern wie Renin und Patient:innenmerkmalen gefolgt von einer gezielten Deeskalation ein vielversprechender Ansatz sein. Die Effektivität dieser Strategien gilt es jedoch noch in randomisierten kontrollierte Studien zu untersuchen.

**Zusatzmaterial online:**

Die Online-Version dieses Beitrags (10.1007/s00063-025-01272-x) enthält eine Liste mit weiterführender Literatur.

## Abstract (en)

Hemodynamic stabilization and preservation of organ perfusion are central elements in the management of septic shock. This is achieved by fluid resuscitation and by administration of vasoactive agents. Current guidelines recommend norepinephrine as the first-line vasoactive substance. In cases of high norepinephrine requirements the addition of nonadrenergic vasopressors is recommended. Furthermore, evidence suggests that early use of complementary vasoactive substances may provide additional benefits. Such a regimen, in terms of a broad-spectrum vasopressor approach, appears physiologically plausible. Post hoc analyses of studies investigating vasopressin or angiotensin II also suggest that specific subphenotypes may particularly benefit from individual vasoactive agents. Adjunctive therapy with hydrocortisone and fludrocortisone can improve vasopressor responsiveness and reduce mortality. In cases of cardiac dysfunction, a trial with dobutamine or a switch from norepinephrine to epinephrine is recommended. To enhance inodilator effects, milrinone or levosimendan may represent additional therapeutic options for certain patients. Although short-acting beta-blockers are not part of the standard treatment for septic shock, they may, in selected cases, contribute to hemodynamic improvement in patients with inadequately high sinus tachycardia or atrial tachyarrhythmias. Based on pathophysiological considerations and the currently available evidence, targeted use of specific vasoactive substances in defined subphenotypes may be justified. An initial broad-spectrum vasopressor strategy incorporating biomarkers such as renin and patient-specific characteristics followed by a focused de-escalation approach could represent a promising concept. However, the effectiveness of these strategies requires further investigation in randomized controlled trials.

## Hintergrund

Das Aufrechterhalten des Kreislaufs ist neben der Infektionskontrolle ein Kernelement in der Therapie des septischen Schocks. Zeigt sich in der Sepsis nach der initialen adäquaten Flüssigkeitsgabe eine persistierende Hypotension und bleibt das Laktat > 2 mmol/l, ist die Definition des septischen Schocks erfüllt, womit die Indikation für den Einsatz vasoaktiver Substanzen gegeben ist. Ab wann die Flüssigkeitsgabe als adäquat angesehen wird und somit eine Therapie mit vasoaktiven Substanzen begonnen werden soll, liegt derzeit im individuellen Ermessensspielraum, zumal in 2 rezenten Studien keine Vor- oder Nachteile einer restriktiven Flüssigkeitsgabe mit entsprechend früherem Einsatz vasoaktiver Substanzen gefunden wurden [1, 2].

## Vasoaktive Standardtherapie

Die Empfehlungen der Surviving Sepsis Campaign (SSC) für vasoaktive Substanzen im septischen Schock beinhalten eine schrittweise Eskalation. In erster Linie wird der Beginn mit Noradrenalin empfohlen. Lässt sich mit niedrigen bis moderaten Noradrenalindosen (üblicherweise 0,25–0,5 µg/kgKG und Minute) kein adäquater mittlerer arterieller Blutdruck (MAP) erreichen, sollte die Zugabe von Vasopressin erwogen werden. Bei kardialer Dysfunktion wird geraten, Dobutamin zu ergänzen oder von Noradrenalin auf Adrenalin zu wechseln. Dieses Vorgehen gilt derzeit als evidenzbasierte vasoaktive Standardtherapie im septischen Schock. Ob und in welchen Fällen ein Abweichen von diesem Schema gerechtfertigt sein könnte, wird im Folgenden ausgeführt.

## Individualisierte Therapie

Die Datenlage für die Standardempfehlungen der SSC-Leitlinien ist überwiegend schwach bis moderat. Darüber hinaus gibt es zunehmend Evidenz, dass bestimmte Subphänotypen der Sepsis von spezifischen Therapiestrategien profitieren könnten. Die derzeit im deutschsprachigem Raum verfügbaren vasoaktiven Substanzen, die im septischen Schock üblicherweise zur Anwendung kommen, umfassen Noradrenalin, Adrenalin, Phenylephrin, Vasopressin, Angiotensin 2 (AT II) sowie kurzwirksame β‑Blocker wie Esmolol oder Landiolol. Als weitere adjunktive Therapiemöglichkeiten zur hämodynamischen Optimierung stehen Hydrokortison und Fludrokortison zur Verfügung. Wesentliche Charakteristika der klassischen Vasopressoren sind in der Tab. 1 dargestellt.

**Tab. 1:** Vasopressoren zur Behandlung des septischen Schocks. (Modifiziert nach [44], © D. De Backer et al. CC BY 4.0, (https://creativecommons.org/licenses/by/4.0/))

Medikament	Rezeptoraktivität	Hämodynamische und organspezifische Effekte	Aspekte für spezifische Patient:innengruppen
Noradrenalin	α_1_-, α_2_-, β_1_- und β_3_-Rezeptor-Agonist	Deutlicher Anstieg des MAP	Erste Wahl für die meisten Patient:innen
		Minimale Erhöhung von CO und HF	
Adrenalin	β_1_-, β_2_- und dosisabhängige α_1_-Rezeptor-Aktivität	Deutlicher Anstieg von MAP und HF	Bei vermuteter anaphylaktischer Komponente Bradykardie im septischen Schock Refraktärer septischer Schock mit myokardialer Dysfunktion
		Moderater Anstieg des CO	
		Tachykardie und Arrhythmien	
		Abnahme der Splanchnikusperfusion bei hohen Dosen	
		Wichtige metabolische Effekte (z. B. Beschleunigung der Glykolyse, thermogene Wirkung, Hypokaliämie)	
Phenylephrin	Reiner α_1_-Agonist	Deutlicher Anstieg des MAP	Falls Noradrenalin nicht verfügbar ist
		Abnahme des CO	
		Abnahme der Splanchnikusperfusion	
Vasopressin	VPR1- und VPR2-Agonist	Deutlicher Anstieg des MAP	Patient:innen mit Arrhythmien Patient:innen mit AKI 0–I Vermeidung bei Patient:innen mit peripherer Ischämie
		Minimale Erhöhung der HF und geringere Inzidenz von Arrhythmien	
		Verbesserung der GFR	
		Erhöhtes Risiko für digitale Nekrosen	
		Potenziell prokoagulatorische Effekte	
Angiotensin II	Angiotensin-I- und Angiotensin-II-Rezeptor	Deutlicher Anstieg des MAP	Patient:innen mit refraktärem septischem Schock Patient:innen mit AKI III, RRT und hohem Reninwert
		Minimaler Anstieg des CO	
		Tachykardie	
		Verbesserung der GFR – geringere RRT-Dauer	
		Erhöhtes Risiko für Lungenembolien	
		Erhöhtes Risiko für Pilzinfektionen	

*AKI* akutes Nierenversagen,* ANG* Angiotensin, *CO* Herzzeitvolumen, *GFR* glomeruläre Filtrationsrate, *HF* Herzfrequenz, *MAP* mittlerer arterieller Blutdruck, *RRT* Nierenersatztherapie, *VPR* Vasopressinrezeptor

### Initialtherapie

Die klare Empfehlung der SSC für Noradrenalin als primäre vasoaktive Substanz im septischen Schock basiert im Wesentlichen auf dem besseren Nebenwirkungsprofil und dem geringeren Preis von Noradrenalin im Vergleich zu anderen vasoaktiven Substanzen. Adrenalin würde mit mehr Tachykardien einhergehen und zudem die Laktatproduktion stimulieren [3]. Ob der Einsatz von Adrenalin anstelle von Noradrenalin als primäre vasoaktive Substanz die Mortalität beeinflussen würde, ist unklar, da eine Vergleichsstudie zwar keinen signifikanten Mortalitätsunterschied zeigte, aber für dieses Outcome nicht ausreichend gepowert war [3]. Im Vergleich zu dem früher verwendeten Dopamin bietet Noradrenalin tatsächlich einen Mortalitätsvorteil und verursacht darüber hinaus weniger Nebenwirkungen, insbesondere weniger Arrhythmien [4].

Für die Verwendung von Vasopressin als First-line-Vasopressor anstatt Noradrenalin gibt es derzeit wenig Evidenz. In der VANISH-Studie zeigte sich lediglich, dass Vasopressin in diesem Setting die Nierenersatztherapierate reduziert [5]. Allerdings erhielten > 80 % der Patient:innen bereits bei der Randomisierung Noradrenalin, sodass die Studie eher die Effekte einer frühen Kombinationstherapie als die von Vasopressin als einem primären Vasopressor zeigt [5]. In der VANCS-II-Studie hatte Vasopressin keinen Mortalitätsvorteil gegenüber Noradrenalin als primäre vasoaktive Substanz [6]. In einer retrospektiven Subphänotypisierung der Patient:innen aus der VASST-Studie wurde bei einem Subtyp des akuten Nierenversagens (AKI), der durch geringer ausgeprägte Inflammation, weniger schweres AKI und geringere Krankheitsschwere charakterisiert war, eine reduzierte Mortalität durch die Behandlung mit Vasopressin nachgewiesen [7].

Auch für die Anwendung von Phenylephrin statt Noradrenalin gibt es keine Evidenz für einen Vorteil. Die Verwendung von Phenylephrin geht zwar mit einer geringeren Herzfrequenz einher, führt jedoch zu einem Laktatanstieg und hat möglicherweise negative Effekte auf die Nierenfunktion und gastrointestinale Perfusion durch eine vermehrte Vasokonstriktion des Splanchnikusgebietes [8, 9]. Es gibt sogar indirekte Hinweise auf eine Verschlechterung des Outcomes bei der Verwendung von Phenylephrin statt Noradrenalin aus einer retrospektiven Analyse einer Zeit, in der es in den USA teilweise zu Lieferengpässen für Noradrenalin kam [10].

Für den Einsatz von AT II als primären Vasopressor gibt es derzeit nur eine prospektive Observationsstudie [11]. Neben einem Signal für eine verbesserte Mortalität auf der Intensivstation (ICU) deuten diese Daten auf mögliche protektive Effekte von AT II auf das Myokard hin. Zudem könnte bei Patient:innen mit vorangegangener Inhibitortherapie des Renin-Angiotensin-Aldosteron-Systems (RAAS) ein zusätzlicher Benefit im Hinblick auf renale Effekte bestehen [11].

Zusammenfassend spricht aus der vorliegenden Evidenz viel für und wenig gegen den Einsatz von Noradrenalin als primäre vasoaktive Substanz, zumindest bis zu einer moderaten Dosierung.

### Kombination vasoaktiver Substanzen – Breitspektrumvasopressoren

**Abb. 1:**
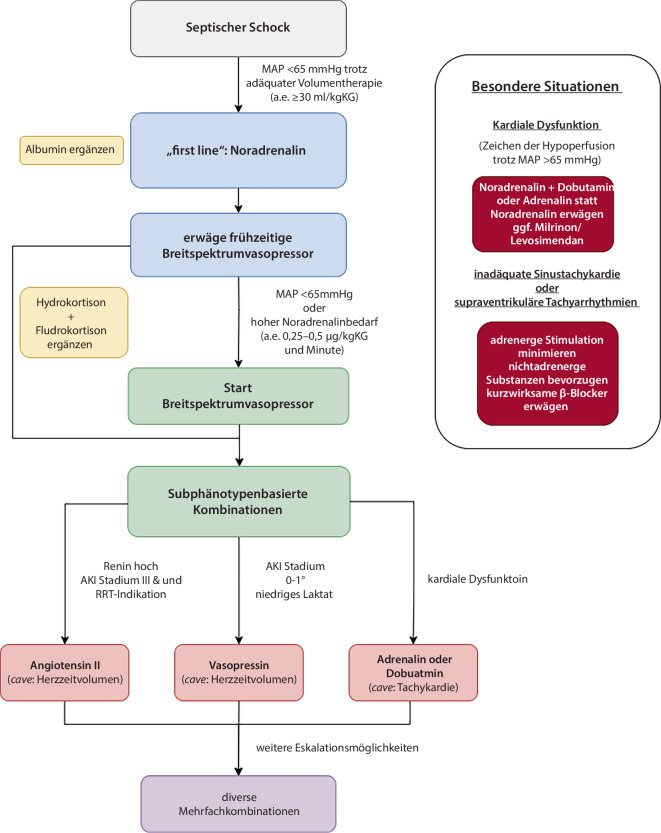
Algorithmus zur Anwendung vasoaktiver Substanzen beim septischen Schock unter Berücksichtigung subphänotypbasierter Strategien. *AKI* akutes Nierenversagen, *MAP* mittlerer arterieller Blutdruck, *RRT* Nierenersatztherapie

Hinsichtlich der weiteren Eskalation der vasoaktiven Therapie gibt es sowohl für den Zeitpunkt als auch für die Substanzwahl weniger klare Evidenz. Die Entwicklung scheint derzeit in Richtung Anwendung eines „early multimodal vasopressor“ [12] oder „Breitsprektrumvasopressor“ Konzept [13] zu gehen (Abb. 1). Es deuten mittlerweile einige Studien darauf hin, dass der frühzeitige Beginn mehrerer komplementärer vasoaktiver Medikamente vorteilhaft sein kann. Die Kombination von Katecholaminen mit nichtkatecholaminergen Vasopressoren im Sinne einer Breitspektrumvasopressortherapie scheint auch aus pathophysiologischer Perspektive vielversprechend zu sein. Schließlich werden Vasotonus und Blutdruck nicht ausschließlich durch das adrenerge System reguliert, sondern unter anderem auch durch das Vasopressinsystem und das RAAS. Jedes dieser 3 Systeme ist im septischen Schock beeinträchtigt und kann gezielt medikamentös moduliert werden. Unterstützende Daten für dieses Konzept gibt es vor allem für die Kombinationen aus Noradrenalin mit Vasopressin oder mit AT II.

#### Kombination von Noradrenalin mit Vasopressin

Im septischen Schock wurden inadäquat niedrige Vasopressinlevel beobachtet [14]. Während in der Initialphase eine Gegenregulation mit erhöhten Vasopressinspiegeln erfolgt, entwickelt etwa ein Drittel der Patient:innen im weiteren Verlauf eine relative Vasopressindefizienz [15]. Ein Grund hierfür könnte unter anderem die inhibitorische Wirkung hoher Noradrenalinlevel auf die Vasopressinfreisetzung sein [14]. Vor diesem Hintergrund erscheint es aus pathophysiologischer und strategischer Sicht sinnvoll, anstatt Noradrenalin exzessiv zu steigern, frühzeitig Vasopressin hinzuzufügen, um Vasopressin auf adäquate Level anzuheben [14]. Bei dieser Strategie wird Vasopressin in niedriger Dosierung vorwiegend im Sinne einer adjunktiven Hormonersatztherapie, denn als hochdosierte Vasopressortherapie eingesetzt.

Eine retrospektive Analyse ergab, dass eine sehr frühe Ergänzung von Noradrenalin um Vasopressin mit einem Mortalitätsbenefit einhergeht [16]. Sehr früh bedeutet bereits bei einer Noradrenalindosis, die deutlich unter dem von der SSC angegebenen üblichen Bereich liegt. Ein solcher Zusammenhang zeigte sich auch in der randomisierten kontrollierten VASST-Studie [17]. Hier brachte die Zugabe von niedrigdosiertem Vasopressin (0,01–0,03 U/min) zu Noradrenalin zwar in der Gesamtpopulation keinen Mortalitätsbenefit, aber in der vordefinierten Subgruppe von Patient:innen mit niedrigem Noradrenalinbedarf (5–14 µg/min; [17]). In Post-hoc-Analysen dieser VASST-Studie fanden sich noch 2 weitere Subgruppen von Patient:innen, die einen Trend zu niedrigerer Mortalität durch die Kombination von Vasopressin und Noradrenalin hatten, diejenige mit niedrigem Laktat (< 1,4 mmol/l [18] und ≤ 2 mmol/l [19]) bzw. diejenige mit einem akuten Nierenversagen (AKI) im Stadium „risk“ nach den Kriterien „risk of renal dysfunction, injury to kidney, failure or loss of kidney function, end-stage kidney disease“ (RIFLE; [20].) Letztere zeigte auch Vorteile in renalen Endpunkten [20]. Ein ähnliches Bild ergab eine Metaanalyse, in der sich Trends zu einem Mortalitätsbenefit durch Vasopressin bei folgenden Subgruppen zeigte:

- früher Beginn von Vasopressin (< 12 h nach Beginn des Schocks),
- niedrige Noradrenalindosis (< 15 µg/min),
- niedriges Laktat (< 2 mmol/l) und
- AKI im Stadium ≤ 1 [21].

Ein Ansprechen auf Vasopressin, charakterisiert durch eine hämodynamische Verbesserung in den ersten 6 h nach Vasopressinbeginn, kann bei rund der Hälfte der Patient:innen beobachtet werden [22, 23] und ist mit einem besseren Outcome assoziiert [24]. Zu den bisher identifizierten Faktoren, die mit einer Vasopressinresponsivität einhergehen, zählen unter anderem ein niedriges Laktat [22], ein höherer arterieller pH-Wert [25] sowie eine höhere linksventrikuläre Ejektionsfraktion [23].

Umgekehrt könnte Vasopressin im Fall eines bereits inadäquat niedrigen Herzzeitvolumens diesen weiter reduzieren [26] und die Verwendung von inotropen Substanzen erforderlich machen [27]. Die hierzu vorliegenden Daten zeigen jedoch kein konsistentes Bild. Darüber hinaus wurden Patient:innen mit chronischer Herzinsuffizienz (New York Heart Association [NYHA] III und IV) in der großen VASST-Studie [17] auch ausgeschlossen. Diese potenzielle Auswirkung auf das Herzzeitvolumen könnte zumindest teilweise indirekt durch den herzfrequenzsenkenden Effekt von Vasopressin vermittelt sein [27], ein Effekt, der bei ausreichender kontraktiler Reserve durchaus vorteilhaft sein könnte. Zusammenfassend bedarf die Anwendung von Vasopressin bei Patient:innen mit eingeschränkter systolischer linksventrikulärer Funktion oder inadäquat niedrigem Herzzeitvolumen besonderer Vorsicht [23], insbesondere bei der Verwendung von höheren Dosierungen [26] oder bei Bolusgaben von Vaspressinrezeptoragonisten wie z. B. Terlipressin [28]. Negative Effekte von Vasopressin auf das Herzzeitvolumen oder von Analoga wie Terlipressin auf die Splanchnikusperfusion [29] könnten insbesondere im Fall einer unzureichenden Volumentherapie verstärkt zum Tragen kommen [30].

#### Kombination von Noradrenalin mit Angiotensin II

Auch für eine Kombination von Noradrenalin und AT II gibt es mittlerweile Evidenz, die vor allem auf Daten der ATHOS-3-Studie basiert [31]. In dieser Studie wurde die Wirksamkeit von AT II als Vasopressor im vasodilatatorischen, überwiegend (80 %) septischen Schock, gezeigt [31]. Die Zugabe von AT II zu einer bereits relativ hohen Noradrenalindosierung (> 0,2 µg/kgKG und Minute) führte zu einem Anstieg des MAP sowie zu einer Reduktion der benötigten Noradrenalindosis [31]. In Post-hoc-Analysen dieser ATHOS-3-Studie wurde untersucht, welche Patient:innen am ehesten von einer Therapie mit AT II profitieren könnten. Ähnlich wie für Vasopressin zeigte sich ein Mortalitätsbenefit durch AT II bei Patient:innen mit niedrigem Noradrenalinbedarf (≤ 0,25 µg/kgKG und Minute; [32]). Eine weitere Subgruppe, die hinsichtlich der MAP-Steigerung besonders gut auf die AT-II-Therapie ansprach und sowohl einen Mortalitätsbenefit als auch ein verbessertes renales Outcome hatte, bildeten Patient:innen, bei denen bereits bei Beginn der AT-II-Therapie ein AKI mit Nierenersatztherapiepflichtigkeit bestand [33]. Dieser Zusammenhang erscheint durchaus plausibel, zumal in der Frühphase des septischen Schocks häufig ein hyperreninämischer Hypoaldosteronismus beobachtet wird, der mit einer erhöhten Inzidenz an AKI und Nierenersatztherapiepflichtigkeit einhergeht [34]. Pathophysiologisch kommt es im septischen Schock zu einer Verminderung von Aldosteron und AT II, was sekundär zu einer Reninfreisetzung führt [34]. In diesem Kontext wird, analog zum Konzept der „critical illness-related corticosteroid insufficiency“ (CIRCI) auch der Begriff der „critical illness-related mineralcorticoid insufficiency“ (CIRMI) verwendet [35]. Dementsprechend ist im septischen Schock die adjunktive Therapie mit Hydrokortison in Kombination mit Fludrokortison sowohl pathophysiologisch plausibel als auch evidenzbasiert gerechtfertigt [36–38]. Damit ist auch das Ergebnis der Post-hoc-Analyse von Bellomo et al., dass nur Patient:innen mit einem hohen Reninlevel (> 173 pg/ml) einen Mortalitätsbenefit durch AT II hatten, kompatibel [39]. Nach der Gabe von AT II kommt es zu einem Abfall der Reninlevel, was zum einen die postulierte Wirkungsweise von AT II unterstützt und andererseits bedeutet, dass Renin als Enrichment-Parameter nur vor der Gabe von AT II bestimmt werden kann [39].

Zur Indikationsstellung von AT II ist außerdem zu beachten, dass in der ATHOS-3-Studie Patient:innen mit niedrigem Herzzeitvolumen ausgeschlossen wurden. Zudem sollte bei der Anwendung von AT II, ähnlich wie bei Vasopressin, besonderes Augenmerk auf potenzielle negative Effekte von AT II auf das Herzzeitvolumen gelegt werden.

### Mehrfachkombinationen

Im schweren septischen Schock ist häufig eine Kombination mehrerer vasoaktiver Substanzen erforderlich. In den aktuellen SSC-Leitlinien wird bei inadäquatem MAP unter einer Kombinationstherapie mit Noradrenalin und Vasopressin die Ergänzung von Adrenalin empfohlen [40]. Im Hinblick auf das Breitspektrumvasopressorkonzept kann auch die Kombination mit anderen Vasopressoren sinnvoll sein. Aussagekräftige Studiendaten liegen für dieses Setting derzeit nicht vor. Obwohl in der ATHOS-3-Studie 70 % der Patient:innen vor Studienbeginn bereits eine Kombination aus Noradrenalin und Vasopressin erhielten, wurde angestrebt, Vasopressin vor Beginn der AT-II-Therapie auszuschleichen [31]. Daher lassen sich aus der ATHOS-3-Studie keine klaren Schlüsse zu dieser spezifischen Fragestellung ziehen. Eine Propensity-score-Analyse retrospektiver Daten deutet darauf hin, dass die Eskalation mit AT II als drittem zusätzlichem Vasopressor im refraktären septischen Schock keinen Mortalitätsbenefit bringt [41]. Auf den ersten Blick scheint dies im Widerspruch zu dem zuvor beschriebenen Benefit von AT II zu stehen. Tatsächlich fügt es sich jedoch gut in das Bild, dass AT II besonders wirksam ist, wenn der Einsatz im Sinne des Breitspektrumvasopressorkonzepts früh im Verlauf des septischen Schocks erfolgt. Diese Interpretation wird auch durch die Daten einer retrospektiven multizentrischen Studie unterstützt, in der die Wahrscheinlichkeit für ein Ansprechen auf AT II bei Patient:innen, die bereits Vasopressin erhielten, 6‑fach höher war und ein Ansprechen auf AT II mit einer niedrigeren Mortalität assoziiert war [42].

### Kardiale Dysfunktion

Der septische Schock geht häufig mit einer myokardialen Dysfunktion einher, die auch durch Vasopressoren induziert oder weiter verschlechtert werden kann, sodass kein adäquates Herzzeitvolumen erreicht wird. Klinisch können persistierende Zeichen der Hypoperfusion trotz eines adäquaten Flüssigkeitsstatus und MAP darauf hinweisen [40]. In solchen Fällen kann der Einsatz inotropiesteigernder Substanzen in Erwägung gezogen werden [40], um das Herzzeitvolumen zu erhöhen. In erster Linie wir die Zugabe von Dobutamin zu Noradrenalin oder die Umstellung von Noradrenalin auf Adrenalin empfohlen [40]. Beide Strategien werden derzeit als gleichwertig erachtet, zumal es keine direkten Vergleichsstudien gibt. Die Wirkungen von Dobutamin im septischen Schock sind komplex und die potenziellen Vorteile theoretisch vielfältig. Eine Zunahme des Schlagvolumenindex (SVI) dürfte sich mit Dobutamin am ehesten bei Patient:innen mit niedriger linksventrikulärer Ejektionsfraktion (LVEF) erreichen lassen [43]. Allerdings lässt sich der Effekt von Dobutamin im Einzelfall nicht zuverlässig abschätzen, weshalb im Fall einer kardialen Dysfunktion ein Dobutaminversuch (initial in einer Dosis von 2,5–5 µg/kgKG und Minute, ggf. bis 20 µg/kgKG und Minute; [44]) sinnvoll sein kann. Ähnlich ist der Einsatz von Adrenalin in dieser Situation zu sehen. Führt die Therapie mit diesen Substanzen jedoch nicht zu einer Verbesserung der Minderperfusion oder treten relevante Nebenwirkungen wie atriale Tachykardien auf, sollten sie trotz ihrer theoretisch vorteilhaften Effekte abgesetzt werden. Dies gilt insbesondere, da ein Mortalitätsbenefit von Dobutamin im septischen Schock bisher nicht nachgewiesen wurde und Observationsstudien eher auf eine möglicherweise erhöhte Mortalität inotroper Substanzen in dieser Situation hindeuten [45]. Von Levosimendan raten die SSC-Leitlinien ab [40], da bisher kein Mortalitätsbenefit durch diese Substanz gezeigt wurde [46, 47], aber die Verwendung unter anderem mit einem erhöhten Risiko für supraventrikuläre Tachykardien einhergeht [47]. Die Empfehlung der SSC gegen Levosimendan basiert jedoch im Wesentlichen auf der LeoPARDS-Studie [47], deren Studiendesign nicht differenziert genug war, um den Stellenwert dieses Inodilatators im septischen Schock im Detail herauszuarbeiten. Möglicherweise wurden jedoch in den vorliegenden Studien die potenziell vorteilhaften Wirkungen von Levosimendan indirekt von den durch die Vasodilatation getriggerten nachteiligen Effekten, wie der resultierenden vermehrten Flüssigkeitsgabe und dem höheren Noradrenalinbedarf, übertroffen.

Andere Autoren schlagen bei kardialer Dysfunktion trotz der begrenzten Evidenzlage eine schrittweise Eskalation inotropiesteigernder Substanzen im Rahmen eines personalisierten hämodynamischen Managements vor [44]: Nach Beginn mit Dobutamin wird im zweiten Schritt empfohlen, PDE3-Hemmer (Enoximon oder Milrinon) entweder ergänzend einzusetzen oder statt Dobutamin zu verwenden. Bei schwerer kardialer Dysfunktion wird im dritten Schritt Levosimendan entweder als Ergänzung oder als Ersatz vorgeschlagen. Dabei sollten gewünschte Effekte und potenzielle Nebenwirkungen fortlaufend beurteilt und die Substanzen jeweils in der niedrigsten wirksamen Dosis verwendet werden [44].

### Tachykardie

Eine Sinustachykardie kann im Rahmen eines septischen Schocks ein adäquater Kompensationsmechanismus, aber auch Ausdruck einer autonomen Dysfunktion oder Folge einer exzessiven exogenen adrenergen Stimulation sein, beispielsweise durch die Verabreichung von Noradrenalin oder Adrenalin. Durch die Tachykardie verkürzt sich die diastolische Füllungszeit, was das Schlagvolumen vermindern kann. Zudem ist Vorhofflimmern im septischen Schock unabhängig von anderen Faktoren mit einer erhöhten Mortalität assoziiert [48]. Dabei scheint Vorhofflimmern nicht nur die Folge eines schweren Krankheitsverlaufs zu sein, sondern auch maßgeblich zu einer weiteren Verschlechterung beizutragen [49]. Daher erscheinen Strategien zur Vermeidung von Vorhofflimmern als vorteilhaft, zumal die negativen hämodynamischen Effekte des Vorhofflimmerns selbst nach der Konversion in den Sinusrhythmus noch über einen längeren Zeitraum persistieren können. Neben β‑Blockern werden häufig auch Antiarrhythmika mit bedenklichem Nebenwirkungspotenzial, wie beispielsweise Amiodaron, zur Frequenz- und Rhythmuskontrolle eingesetzt [48].

Bei einer Neigung zu Tachykardien, sei es in Form einer inadäquat hohen Sinustachykardie oder anderer supraventrikulärer Tachyarrhythmien, kann die Kombination von Noradrenalin mit nichtadrenerg wirkenden vasokonstriktorischen Substanzen, wie Vasopressin oder AT II, sinnvoll sein, um die zusätzliche adrenerge Stimulation möglichst zu minimieren [40]. Vasopressin beispielsweise kann die Herzfrequenz um etwa 10 % senken [17, 50]. Bei septischen Patient:innen mit inadäquat hoher Herzfrequenz oder Tachyarrhythmien kann die Anwendung eines β‑Blockers eine hämodynamische Verbesserung bewirken und unerwünschte Wirkungen endogener und exogener Katecholamine reduzieren. In hämodynamisch kritischen Situationen bietet sich vor allem der Einsatz kurzwirksamer und selektiver β‑Blocker, wie Esmolol oder Landiolol, an. Bereits im Jahr 2013 zeigten Morelli et al., dass die Kombination von Esmolol und Milrinon die Mortalität als sekundären Endpunkt signifikant von 80,5 auf 49,9 % senkte [51]. Eine randomisierte kontrollierte Studie wies zudem nach, dass Landiolol die Häufigkeit neu auftretender Arrhythmien von 25 auf 9 % reduziert [52]. Darüber hinaus legen Daten aus einer kleinen retrospektiven Studie nahe, dass die Verwendung von Landiolol bei supraventrikulären Tachyarrhythmien mit einer höheren Konversionsrate in den Sinusrhythmus assoziiert ist [53].

Die Verwendung von kurzwirksamen β‑Blockern gehört derzeit nicht zur Standardtherapie im septischen Schock. Unter bestimmten Umständen kann ein Einsatz jedoch gerechtfertigt sein. Da die Effekte einer β‑Blockade nicht sicher vorhergesagt werden können, sollte bei unerwünschten Wirkungen oder einer hämodynamischen Verschlechterung – beispielsweise einer Zunahme des Noradrenalinbedarfs [54], einer Erhöhung des Laktatspiegels oder einem Abfall der zentralvenösen Sättigung (ScvO_2_) – die Dosierung angepasst oder die β‑Blocker-Gabe beendet werden. Ein potenziell vorteilhaftes Regime könnte insbesondere die Kombination kurzwirksamer β‑Blocker mit inodilatatorisch wirksamen Substanzen darstellen.

Es gilt, in künftigen Studien noch herauszufinden, welche Patient:innen potenziell von der β‑Blocker-Anwendung profitieren könnten und inwiefern eine Kombination mit Inodilatatoren zusätzlichen Nutzen bietet [55].

## Resümee

Faktoren, die das Ansprechen und den Effekt einzelner vasoaktiver Substanzen beeinflussen – wie genetische Polymorphismen, Patientencharakteristika, Krankheitsstadium oder die Art des Pathogens – sind bisher unzureichend erforscht. Die derzeit vorliegende Evidenz ermöglicht jedoch bereits im klinischen Alltag die Anwendung subphänotypbasierter Strategien.

Die Rolle von Noradrenalin als primäre vasoaktive Substanz ist gut etabliert. Allerdings deuten das Nebenwirkungsprofil bei höheren Noradrenalindosierungen sowie die Datenlage darauf hin, dass die Ergänzung zusätzlicher vasoaktiver Substanzen einer fortlaufenden Dosiseskalation von Noradrenalin vorzuziehen ist. Für die weitere Eskalation liegt derzeit keine überzeugende Evidenz vor, die ein allgemeines Standardschema mit einer spezifischen vasoaktiven Substanz rechtfertigen würde. Allerdings können aus Post-hoc-Analysen und pathophysiologischen Überlegungen subphänotypbasierte Strategien abgeleitet werden. Auch wenn hierfür randomisierte kontrollierte Studien bislang fehlen, kann der gezielte Einsatz spezifischer vasoaktiver Substanzen bei bestimmten Subphänotypen gerechtfertigt sein. Darüber hinaus könnte eine initiale Breitspektrumvasopressortherapie, die spezifische Biomarker wie Renin und Patient:innencharakteristika berücksichtigt, gefolgt von einer gezielten Deeskalation, ähnlich wie bei der antiinfektiven Therapie, ein vielversprechender Ansatz sein [13, 56]. Die Effektivität dieser Strategie ist jedoch bisher nicht durch randomisiert-kontrollierte Studien belegt.

Angesichts der Komplexität der Sepsis erfordert eine individualisierte vasoaktive Therapie ein umfassendes hämodynamisches Monitoring, das neben kardialen Parametern, wie Herzzeitvolumen, SVI, „stroke work index“ (SWI) und systemischer vaskulärer Widerstandsindex (SVRI), auch Enrichment-Parameter (z. B. Renin) sowie biochemische Marker, wie Laktat, ScvO_2_ und die Differenz zwischen zentralvenösem und arteriellem pCO_2_ (pV – aCO_2_, Delta-CO_2_), einbezieht. Anstelle standardisierte Therapieziele (wie z. B. eines vorgegebenen Herzfrequenzziels bei β‑Blockern) zu verwenden, empfiehlt es sich zudem, die Auswirkungen einzelner Therapieschritte auf die genannten Parameter sowie auf den Gesamtzustand der Patient:innen systematisch zu evaluieren und in die weitere Therapieoptimierung einfließen zu lassen.

## Fazit für die Praxis

- Nach einer adäquaten Volumentherapie wird der Einsatz von Noradrenalin als primäre vasoaktive Substanz empfohlen.
- Wann die Volumentherapie als adäquat anzusehen ist, bedarf einer individuellen Einschätzung.
- Bei hohem Noradrenalinbedarf könnte es vorteilhafter sein, andere vasoaktive Substanzen zu ergänzen, anstatt die Noradrenalindosis weiter zu steigern. Eine frühzeitige Kombinationstherapie im Sinne einer Breitspektrumvasopressortherapie könnte hierbei von Vorteil sein.
- Patient:innen mit niedrigem Laktat und ohne akutes Nierenversagen (AKI) oder mit AKI I scheinen geeignete Kandidat:innen für den Einsatz von Vasopressin zu sein, während Patient:innen mit AKI III, die eine Nierenersatztherapie (RRT) benötigen und hohe Reninlevel aufweisen, eher von einem AT-II-Einsatz zu profitieren scheinen.
- Die adjunktive Therapie mit Hydrokortison und Fludrokortison kann vorteilhafte hämodynamische Effekte bringen.
- Bei kardialer Dysfunktion kann eine Ergänzung mit Dobutamin versucht werden. Gegebenenfalls kann, z. B. bei Erfolglosigkeit von Inotropika, unter Berücksichtigung der kardialen Funktion auch eine Umstellung von Noradrenalin auf Adrenalin erwogen werden. Auch Inodilatatoren, wie Levosimendan oder Milrinon, können in Einzelfällen erwogen werden.
- Bei Patient:innen mit inadäquater Sinustachykardie oder supraventrikulären Arrhythmien kann ein Therapieversuch mit kurzwirksamen β‑Blockern gerechtfertigt sein.
- In hämodynamisch komplexen Situationen empfiehlt es sich, neben einem erweiterten hämodynamischen Monitoring zusätzliche Parameter, wie die zentralvenöse Sättigung (ScvO_2_), die Differenz zwischen zentralvenösem und arteriellem pCO_2_ (pV – aCO_2_, Delta-CO_2_) sowie die Echokardiographie in die Entscheidungsfindung und die Evaluierung der Therapieeffekte einzubeziehen.

## Supplementary Information

ESM 1: Weiterführende Literatur

## References

[1] National Heart L, Blood Institute P, Early Treatment of Acute Lung Injury Clinical Trials N, Shapiro NI, Douglas IS, Brower RG et al (2023) Early restrictive or liberal fluid management for sepsis-induced hypotension. N Engl J Med 388(6):499–510 36688507 10.1056/NEJMoa2212663 PMC10685906

[2] Meyhoff TS, Hjortrup PB, Wetterslev J, Sivapalan P, Laake JH, Cronhjort M et al (2022) Restriction of intravenous fluid in ICU patients with septic shock. N Engl J Med. 10.1016/j.jcrc.2024.154737 35709019 10.1056/NEJMoa2202707

[3] Myburgh JA, Higgins A, Jovanovska A, Lipman J, Ramakrishnan N, Santamaria J et al (2008) A comparison of epinephrine and norepinephrine in critically ill patients. Intensive Care Med 34(12):2226–2234 18654759 10.1007/s00134-008-1219-0

[4] Avni T, Lador A, Lev S, Leibovici L, Paul M, Grossman A (2015) Vasopressors for the treatment of septic shock: systematic review and meta-analysis. PLoS ONE 10(8):e129305 26237037 10.1371/journal.pone.0129305 PMC4523170

[5] Gordon AC, Mason AJ, Thirunavukkarasu N, Perkins GD, Cecconi M, Cepkova M et al (2016) Effect of early vasopressin vs norepinephrine on kidney failure in patients with septic shock: the VANISH randomized clinical trial. JAMA 316(5):509–518 27483065 10.1001/jama.2016.10485

[6] Hajjar LA, Zambolim C, Belletti A, de Almeida JP, Gordon AC, Oliveira G et al (2019) Vasopressin versus norepinephrine for the management of septic shock in cancer patients: the VANCS II randomized clinical trial. Crit Care Med 47(12):1743–1750 31609774 10.1097/CCM.0000000000004023

[7] Bhatraju PK, Zelnick LR, Herting J, Katz R, Mikacenic C, Kosamo S et al (2019) Identification of acute kidney injury subphenotypes with differing molecular signatures and responses to vasopressin therapy. Am J Respir Crit Care Med 199(7):863–872 30334632 10.1164/rccm.201807-1346OC PMC6444649

[8] Morelli A, Ertmer C, Rehberg S, Lange M, Orecchioni A, Laderchi A et al (2008) Phenylephrine versus norepinephrine for initial hemodynamic support of patients with septic shock: a randomized, controlled trial. Crit Care 12(6):R143 19017409 10.1186/cc7121 PMC2646303

[9] Morelli A, Lange M, Ertmer C, Dunser M, Rehberg S, Bachetoni A et al (2008) Short-term effects of phenylephrine on systemic and regional hemodynamics in patients with septic shock: a crossover pilot study. Shock 29(4):446–451 17885646 10.1097/shk.0b013e31815810ff

[10] Vail E, Gershengorn HB, Hua M, Walkey AJ, Rubenfeld G, Wunsch H (2017) Association between US norepinephrine shortage and mortality among patients with septic shock. JAMA 317(14):1433–1442 28322415 10.1001/jama.2017.2841

[11] See EJ, Clapham C, Liu J, Khasin M, Liskaser G, Chan JW et al (2023) A pilot study of angiotensin ii as primary vasopressor in critically ill adults with vasodilatory hypotension: the Aramis study. Shock 59(5):691–696 36930693 10.1097/SHK.0000000000002109

[12] Wieruszewski PM, Khanna AK (2022) Early multimodal vasopressors-are we ready for it? Crit Care Med 50(4):705–708 35311781 10.1097/CCM.0000000000005344

[13] Chawla LS, Ostermann M, Forni L, Tidmarsh GF (2019) Broad spectrum vasopressors: a new approach to the initial management of septic shock? Crit Care 23(1):124 30992045 10.1186/s13054-019-2420-y PMC6469125

[14] Holmes CL, Patel BM, Russell JA, Walley KR (2001) Physiology of vasopressin relevant to management of septic shock. Chest 120(3):989–1002 11555538 10.1378/chest.120.3.989

[15] Sharshar T, Blanchard A, Paillard M, Raphael JC, Gajdos P, Annane D (2003) Circulating vasopressin levels in septic shock. Crit Care Med 31(6):1752–1758 12794416 10.1097/01.CCM.0000063046.82359.4A

[16] Sacha GL, Lam SW, Wang L, Duggal A, Reddy AJ, Bauer SR (2022) Association of catecholamine dose, lactate, and shock duration at vasopressin initiation with mortality in patients with septic shock. Crit Care Med 50(4):614–623 34582425 10.1097/CCM.0000000000005317

[17] Russell JA, Walley KR, Singer J, Gordon AC, Hebert PC, Cooper DJ et al (2008) Vasopressin versus norepinephrine infusion in patients with septic shock. N Engl J Med 358(9):877–887 18305265 10.1056/NEJMoa067373

[18] Wacharasint P, Nakada TA, Boyd JH, Russell JA, Walley KR (2012) Normal-range blood lactate concentration in septic shock is prognostic and predictive. Shock 38(1):4–10 22552014 10.1097/SHK.0b013e318254d41a

[19] Russell JA, Lee T, Singer J, Boyd JH, Walley KR (2017) The septic shock 3.0 definition and trials: a vasopressin and septic shock trial experience. Crit Care Med 45(6):940–948 28333757 10.1097/CCM.0000000000002323

[20] Gordon AC, Russell JA, Walley KR, Singer J, Ayers D, Storms MM et al (2010) The effects of vasopressin on acute kidney injury in septic shock. Intensive Care Med 36(1):83–91 19841897 10.1007/s00134-009-1687-x

[21] Nagendran M, Russell JA, Walley KR, Brett SJ, Perkins GD, Hajjar L et al (2019) Vasopressin in septic shock: an individual patient data meta-analysis of randomised controlled trials. Intensive Care Med 45(6):844–855 31062052 10.1007/s00134-019-05620-2

[22] Sacha GL, Lam SW, Duggal A, Torbic H, Bass SN, Welch SC et al (2018) Predictors of response to fixed-dose vasopressin in adult patients with septic shock. Ann Intensive Care 8(1):35 29511951 10.1186/s13613-018-0379-5 PMC5840112

[23] Dugar S, Siuba MT, Sacha GL, Sato R, Moghekar A, Collier P et al (2023) Echocardiographic profiles and hemodynamic response after vasopressin initiation in septic shock: a cross-sectional study. J Crit Care 76:154298 37030157 10.1016/j.jcrc.2023.154298 PMC10239343

[24] Bauer SR, Sacha GL, Siuba MT, Wang L, Wang X, Scheraga RG et al (2023) Vasopressin response and clinical trajectory in septic shock patients. J Intensive Care Med 38(3):273–279 36062611 10.1177/08850666221118282 PMC10236982

[25] Bauer SR, Sacha GL, Siuba MT, Lam SW, Reddy AJ, Duggal A et al (2022) Association of arterial pH with hemodynamic response to vasopressin in patients with septic shock: an observational cohort study. Crit Care Explor 4(2):e634 35156051 10.1097/CCE.0000000000000634 PMC8826954

[26] Holmes CL, Walley KR, Chittock DR, Lehman T, Russell JA (2001) The effects of vasopressin on hemodynamics and renal function in severe septic shock: a case series. Intensive Care Med 27(8):1416–1421 11511958 10.1007/s001340101014

[27] Gordon AC, Wang N, Walley KR, Ashby D, Russell JA (2012) The cardiopulmonary effects of vasopressin compared with norepinephrine in septic shock. Chest 142(3):593–605 22518026 10.1378/chest.11-2604

[28] Morelli A, Ertmer C, Lange M, Dunser M, Rehberg S, Van Aken H et al (2008) Effects of short-term simultaneous infusion of dobutamine and terlipressin in patients with septic shock: the DOBUPRESS study. Br J Anaesth 100(4):494–503 18308741 10.1093/bja/aen017

[29] Asfar P, Pierrot M, Veal N, Moal F, Oberti F, Croquet V et al (2003) Low-dose terlipressin improves systemic and splanchnic hemodynamics in fluid-challenged endotoxic rats. Crit Care Med 31(1):215–220 12545018 10.1097/00003246-200301000-00033

[30] Morelli A, Ertmer C, Rehberg S, Lange M, Orecchioni A, Cecchini V et al (2009) Continuous terlipressin versus vasopressin infusion in septic shock (TERLIVAP): a randomized, controlled pilot study. Crit Care 13(4):R130 19664253 10.1186/cc7990 PMC2750187

[31] Khanna A, English SW, Wang XS, Ham K, Tumlin J, Szerlip H et al (2017) Angiotensin II for the treatment of vasodilatory shock. N Engl J Med 377(5):419–430 28528561 10.1056/NEJMoa1704154

[32] Wieruszewski PM, Bellomo R, Busse LW, Ham KR, Zarbock A, Khanna AK et al (2023) Initiating angiotensin II at lower vasopressor doses in vasodilatory shock: an exploratory post-hoc analysis of the ATHOS‑3 clinical trial. Crit Care 27(1):175 37147690 10.1186/s13054-023-04446-1 PMC10163684

[33] Tumlin JA, Murugan R, Deane AM, Ostermann M, Busse LW, Ham KR et al (2018) Outcomes in patients with vasodilatory shock and renal replacement therapy treated with intravenous angiotensin II. Crit Care Med 46(6):949–957 29509568 10.1097/CCM.0000000000003092 PMC5959265

[34] du Cheyron D, Lesage A, Daubin C, Ramakers M, Charbonneau P (2003) Hyperreninemic hypoaldosteronism: a possible etiological factor of septic shock-induced acute renal failure. Intensive Care Med 29(10):1703–1709 14551679 10.1007/s00134-003-1986-6

[35] Nethathe GD, Lipman J, Anderson R, Feldman C (2022) CIRMI—a new term for a concept worthy of further exploration: a narrative review. Ann Transl Med 10(11):646 35813323 10.21037/atm-21-5572 PMC9263790

[36] Bosch NA, Teja B, Law AC, Pang B, Jafarzadeh SR, Walkey AJ (2023) Comparative effectiveness of fludrocortisone and hydrocortisone vs hydrocortisone alone among patients with septic shock. JAMA Intern Med 183(5):451–459 36972033 10.1001/jamainternmed.2023.0258 PMC10043800

[37] Annane D, Renault A, Brun-Buisson C, Megarbane B, Quenot JP, Siami S et al (2018) Hydrocortisone plus fludrocortisone for adults with septic shock. N Engl J Med 378(9):809–818 29490185 10.1056/NEJMoa1705716

[38] Annane D, Sebille V, Charpentier C, Bollaert PE, Francois B, Korach JM et al (2002) Effect of treatment with low doses of hydrocortisone and fludrocortisone on mortality in patients with septic shock. JAMA 288(7):862–871 12186604 10.1001/jama.288.7.862

[39] Bellomo R, Forni LG, Busse LW, McCurdy MT, Ham KR, Boldt DW et al (2020) Renin and survival in patients given angiotensin II for catecholamine-resistant vasodilatory shock. A clinical trial. Am J Respir Crit Care Med 202(9):1253–1261 32609011 10.1164/rccm.201911-2172OC PMC7605187

[40] Evans L, Rhodes A, Alhazzani W, Antonelli M, Coopersmith CM, French C et al (2021) Surviving sepsis campaign: international guidelines for management of sepsis and septic shock 2021. Intensive Care Med 47(11):1181–1247 34599691 10.1007/s00134-021-06506-y PMC8486643

[41] Quan M, Cho N, Bushell T, Mak J, Nguyen N, Litwak J et al (2022) Effectiveness of angiotensin II for catecholamine refractory septic or distributive shock on mortality: a propensity score weighted analysis of real-world experience in the medical ICU. Crit Care Explor 4(1):e623 35072084 10.1097/CCE.0000000000000623 PMC8769135

[42] Wieruszewski PM, Wittwer ED, Kashani KB, Brown DR, Butler SO, Clark AM et al (2021) Angiotensin II infusion for shock: a multicenter study of postmarketing use. Chest 159(2):596–605 32882250 10.1016/j.chest.2020.08.2074 PMC7856533

[43] Enrico C, Kanoore Edul VS, Vazquez AR, Pein MC, Pérez de la Hoz RA, Ince C et al (2012) Systemic and microcirculatory effects of dobutamine in patients with septic shock. J Crit Care 27(6):630–638 23084135 10.1016/j.jcrc.2012.08.002

[44] De Backer D, Cecconi M, Chew MS, Hajjar L, Monnet X, Ospina-Tascón GA et al (2022) A plea for personalization of the hemodynamic management of septic shock. Crit Care 26(1):372 36457089 10.1186/s13054-022-04255-y PMC9714237

[45] Sato R, Ariyoshi N, Hasegawa D, Crossey E, Hamahata N, Ishihara T et al (2021) Effects of Inotropes on the mortality in patients with septic shock. J Intensive Care Med 36(2):211–219 31793373 10.1177/0885066619892218

[46] Ge Z, Gao Y, Lu X, Yu S, Qin M, Gong C et al (2024) The association between levosimendan and mortality in patients with sepsis or septic shock: a systematic review and meta-analysis. Eur J Emerg Med 31(2):90–97 38015719 10.1097/MEJ.0000000000001105 PMC10901220

[47] Gordon AC, Perkins GD, Singer M, McAuley DF, Orme RM, Santhakumaran S et al (2016) Levosimendan for the prevention of acute organ dysfunction in sepsis. N Engl J Med 375(17):1638–1648 27705084 10.1056/NEJMoa1609409

[48] Klein Klouwenberg PM, Frencken JF, Kuipers S, Ong DS, Peelen LM, van Vught LA et al (2017) Incidence, predictors, and outcomes of new-onset atrial fibrillation in critically ill patients with sepsis. A cohort study. Am J Respir Crit Care Med 195(2):205–211 27467907 10.1164/rccm.201603-0618OC

[49] Walkey AJ, McManus D (2017) When rhythm changes cause the blues: new-onset atrial fibrillation during sepsis. Am J Respir Crit Care Med 195(2):152–154 28084820 10.1164/rccm.201608-1617ED PMC5394791

[50] Russell JA (2019) Vasopressor therapy in critically ill patients with shock. Intensive Care Med 45(11):1503–1517 31646370 10.1007/s00134-019-05801-z

[51] Morelli A, Ertmer C, Westphal M, Rehberg S, Kampmeier T, Ligges S et al (2013) Effect of heart rate control with esmolol on hemodynamic and clinical outcomes in patients with septic shock: a randomized clinical trial. JAMA 310(16):1683–1691 24108526 10.1001/jama.2013.278477

[52] Kakihana Y, Nishida O, Taniguchi T, Okajima M, Morimatsu H, Ogura H et al (2020) Efficacy and safety of landiolol, an ultra-short-acting β1-selective antagonist, for treatment of sepsis-related tachyarrhythmia (J-Land 3S): a multicentre, open-label, randomised controlled trial. Lancet Respir Med 8(9):863–872 32243865 10.1016/S2213-2600(20)30037-0

[53] Okajima M, Takamura M, Taniguchi T (2015) Landiolol, an ultra-short-acting beta1-blocker, is useful for managing supraventricular tachyarrhythmias in sepsis. World J Crit Care Med 4(3):251–257 26261777 10.5492/wjccm.v4.i3.251 PMC4524822

[54] Duska F, Rehberg S (2024) Personalizing beta-blockade in septic shock: finding the right rhythm and rate for the right patient. Intensive Care Med. 10.1007/s00134-024-07731-x 39589504 10.1007/s00134-024-07731-x

[55] Morelli A, Romano SM, Sanfilippo F, Santonocito C, Frati G, Chiostri M et al (2020) Systolic-dicrotic notch pressure difference can identify tachycardic patients with septic shock at risk of cardiovascular decompensation following pharmacological heart rate reduction. Br J Anaesth 125(6):1018–1024 32690246 10.1016/j.bja.2020.05.058

[56] Wieruszewski PM, Khanna AK (2022) Vasopressor choice and timing in vasodilatory shock. Crit Care 26(1):76 35337346 10.1186/s13054-022-03911-7 PMC8957156

