# Supplementary material for: Vasoactive agents in septic shock—individualized strategies
Source: Med Klin Intensivmed Notfmed. 2025 Apr 24;120(5):369–78. [Article in German] doi: 10.1007/s00063-025-01272-x (PMC12106132; doi:10.1007/s00063-025-01272-x)
Supplement: Supplementary file 1 — ESM 1: Weiterführende Literatur [file 63_2025_1272_MOESM1_ESM.pdf]

Weiterführende Literatur:

Alam, A., W. Sovic, J. Gill, N. Ragula, M. Salem, G. J. Hughes, G. B. Colbert and J. L. Mooney (2022). "Angiotensin II: A Review of Current Literature." J Cardiothorac Vasc Anesth 36(4): 1180-1187.

Bellomo, R., L. G. Forni, L. W. Busse, M. T. McCurdy, K. R. Ham, D. W. Boldt, J. Hastbacka, A. K. Khanna, T. E. Albertson, J. Tumlin, K. Storey, D. Handisides, G. F. Tidmarsh, L. S. Chawla and M. Ostermann (2020). "Renin and Survival in Patients Given Angiotensin II for Catecholamine-Resistant Vasodilatory Shock. A Clinical Trial." Am J Respir Crit Care Med 202(9): 1253-1261.

Busse, L. W., X. S. Wang, D. M. Chalikonda, K. W. Finkel, A. K. Khanna, H. M. Szerlip, D. Yoo, S. L. Dana and L. S. Chawla (2017). "Clinical Experience With IV Angiotensin II Administration: A Systematic Review of Safety." Crit Care Med 45(8): 1285-1294.

Chang, W., J. F. Xie, J. Y. Xu and Y. Yang (2018). "Effect of levosimendan on mortality in severe sepsis and septic shock: a meta-analysis of randomised trials." BMJ Open 8(3): e019338.

Cunha-Goncalves, D., V. Perez-de-Sa, A. Larsson, J. Thorne and S. Blomquist (2009). "Inotropic support during experimental endotoxemic shock: part II. A comparison of levosimendan with dobutamine." Anesth Analg 109(5): 1576-1583.

Day, T. A., J. C. Randle and L. P. Renaud (1985). "Opposing alpha- and beta-adrenergic mechanisms mediate dose-dependent actions of noradrenaline on supraoptic vasopressin neurones in vivo." Brain Res 358(1-2): 171-179.

Dubin, A., B. Lattanzio and L. Gatti (2017). "The spectrum of cardiovascular effects of dobutamine - from healthy subjects to septic shock patients." Rev Bras Ter Intensiva 29(4): 490-498.

Dunser, M. W., A. J. Mayr, H. Ulmer, H. Knotzer, G. Sumann, W. Pajk, B. Friesenecker and W. R. Hasibeder (2003). "Arginine vasopressin in advanced vasodilatory shock: a prospective, randomized, controlled study." Circulation 107(18): 2313-2319.

Gordon, A. C., N. Wang, K. R. Walley, D. Ashby and J. A. Russell (2012). "The cardiopulmonary effects of vasopressin compared with norepinephrine in septic shock." Chest 142(3): 593-605.

Landry, D. W., H. R. Levin, E. M. Gallant, R. C. Ashton, Jr., S. Seo, D. D'Alessandro, M. C. Oz and J. A. Oliver (1997). "Vasopressin deficiency contributes to the vasodilation of septic shock." Circulation 95(5): 1122-1125.

Lauzier, F., B. Levy, P. Lamarre and O. Lesur (2006). "Vasopressin or norepinephrine in early hyperdynamic septic shock: a randomized clinical trial." Intensive Care Med 32(11): 1782-1789.

Legrand, M., A. K. Khanna, M. Ostermann, Y. Kotani, R. Ferrer, M. Girardis, M. Leone, G. DePascale, P. Pickkers, P. Tissieres, F. Annoni, K. Kotfis, G. Landoni, A. Zarbock, P. M. Wieruszewski, D. De Backer, J. L. Vincent and R. Bellomo (2024). "The renin-angiotensin-aldosterone-system in sepsis and its clinical modulation with exogenous angiotensin II." Crit Care 28(1): 389.

Malay, M. B., R. C. Ashton, Jr., D. W. Landry and R. N. Townsend (1999). "Low-dose vasopressin in the treatment of vasodilatory septic shock." J Trauma 47(4): 699-703; discussion 703-695.

Morelli, A., C. Ertmer, S. Rehberg, M. Lange, A. Orecchioni, V. Cecchini, A. Bachetoni, M. D'Alessandro, H. Van Aken, P. Pietropaoli and M. Westphal (2009). "Continuous terlipressin versus vasopressin infusion in septic shock (TERLIVAP): a randomized, controlled pilot study." Crit Care 13(4): R130.

O'Neill, P. G., P. R. Puleo, R. Bolli and R. Rokey (1990). "Return of atrial mechanical function following electrical conversion of atrial dysrhythmias." Am Heart J 120(2): 353-359.

Patel, B. M., D. R. Chittock, J. A. Russell and K. R. Walley (2002). "Beneficial effects of short-term vasopressin infusion during severe septic shock." Anesthesiology 96(3): 576-582.

Reuter, D. A., J. A. Russell and A. Mekontso Dessap (2016). "Beta-blockers in septic shock to optimize hemodynamics? Yes." Intensive Care Med 42(10): 1607-1609.

Rudiger, A. and M. Singer (2007). "Mechanisms of sepsis-induced cardiac dysfunction." Crit Care Med 35(6): 1599-1608.

Russell, J. A. (2019). "Vasopressor therapy in critically ill patients with shock." Intensive Care Med 45(11): 1503-1517.

Sato, R., N. Ariyoshi, D. Hasegawa, E. Crossey, N. Hamahata, T. Ishihara, M. Nasu and G. Devendra (2021). "Effects of Inotropes on the Mortality in Patients With Septic Shock." J Intensive Care Med 36(2): 211-219.

Schaich, C. L., D. E. Leisman, M. B. Goldberg, M. R. Filbin, A. K. Khanna and M. C. Chappell (2024). "Dysfunction of the renin-angiotensin-aldosterone system in human septic shock." Peptides 176: 171201.

Singer, M. (2007). "Catecholamine treatment for shock--equally good or bad?" Lancet 370(9588): 636-637.

Wang, Z., Q. Wu, X. Nie, J. Guo and C. Yang (2015). "Combination therapy with milrinone and esmolol for heart protection in patients with severe sepsis: a prospective, randomized trial." Clin Drug Investig 35(11): 707-716.
